# Dementia risk communication. A user manual for Brain Health Services—part 3 of 6

**DOI:** 10.1186/s13195-021-00840-5

**Published:** 2021-10-11

**Authors:** Leonie N. C. Visser, Carolina Minguillon, Gonzalo Sánchez-Benavides, Marc Abramowicz, Daniele Altomare, Karine Fauria, Giovanni B. Frisoni, Jean Georges, Federica Ribaldi, Philip Scheltens, Jetske van der Schaar, Marissa Zwan, Wiesje M. van der Flier, José Luis Molinuevo

**Affiliations:** 1 Alzheimer Center Amsterdam, Department of Neurology, Amsterdam Neuroscience, Vrije Universiteit Amsterdam, Amsterdam UMC, Amsterdam, The Netherlands; 2 Center for Alzheimer Research, Division of Clinical Geriatrics, Department of Neurobiology, Care Sciences and Society, Karolinska Institutet, Solna, Sweden; 3 Barcelonaβeta Brain Research Center (BBRC), Pasqual Maragall Foundation, Barcelona, Spain; 4 IMIM (Hospital del Mar Medical Research Institute), Barcelona, Spain; 5 Centro de Investigación Biomédica en Red de Fragilidad y Envejecimiento Saludable (CIBERFES), Madrid, Spain; 6 Division of Genetic Medicine, Department of Diagnostics, Geneva University Hospitals and University of Geneva, Geneva, Switzerland; 7 Laboratory of Neuroimaging of Aging (LANVIE), University of Geneva, Geneva, Switzerland; 8 Memory Clinic, Geneva University Hospitals, Geneva, Switzerland; 9 Alzheimer Europe, Luxembourg, Luxembourg; 10 Laboratory of Alzheimer’s Neuroimaging and Epidemiology (LANE), Saint John of God Clinical Research Centre, Brescia, Italy; 11 Department of Molecular and Translational Medicine, University of Brescia, Brescia, Italy; 12 Department of Epidemiology and Data Science, Vrije Universiteit Amsterdam, Amsterdam UMC, Amsterdam, The Netherlands

**Keywords:** Brain Health Services, Dementia, Aging, Alzheimer’s disease, Prevention, Risk communication

## Abstract

Growing evidence suggests dementia incidence can be reduced through prevention programs targeting risk factors. To accelerate the implementation of such prevention programs, a new generation of brain health services (BHS) is envisioned, involving risk profiling, risk communication, risk reduction, and cognitive enhancement. The purpose of risk communication is to enable individuals at risk to make informed decisions and take action to protect themselves and is thus a crucial step in tailored prevention strategies of the dementia incidence. However, communicating about dementia risk is complex and challenging.

In this paper, we provide an overview of (i) perspectives on communicating dementia risk from an ethical, clinical, and societal viewpoint; (ii) insights gained from memory clinical practice; (iii) available evidence on the impact of disclosing *APOE* and Alzheimer’s disease biomarker test results gathered from clinical trials and observational studies; (iv) the value of established registries in light of BHS; and (v) practical recommendations regarding effective strategies for communicating about dementia risk.

In addition, we identify challenges, i.e., the current lack of evidence on *what* to tell on an individual level—the actual risk—and on *how* to optimally communicate about dementia risk, especially concerning worried yet cognitively unimpaired individuals. Ideally, dementia risk communication strategies should maximize the desired impact of risk information on individuals’ understanding of their health/disease status and risk perception and minimize potential harms. More research is thus warranted on the impact of dementia risk communication, to (1) evaluate the merits of different approaches to risk communication on outcomes in the cognitive, affective and behavioral domains, (2) develop an evidence-based, harmonized dementia risk communication protocol, and (3) develop e-tools to support and promote adherence to this protocol in BHSs.

Based on the research reviewed, we recommend that dementia risk communication should be precise; include the use of absolute risks, visual displays, and time frames; based on a process of shared decision-making; and address the inherent uncertainty that comes with any probability.

**Supplementary Information:**

The online version contains supplementary material available at 10.1186/s13195-021-00840-5.

## Background

Encouraging evidence suggests dementia incidence can be reduced by means of precision prevention programs targeting risk factors [1, 2]. This entails tailoring risk reduction to the clinical, biological, genetic, and psychosocial characteristics of each patient. To accelerate the implementation of such a precision approach in dementia prevention, a new generation of Brain Health Services (BHSs) can be envisioned [3], guided by risk profiling [4], and risk communication (the present paper), with the general goal of personalized risk reduction [5] and cognitive enhancement [6], in an ethical and equitable context [7].

The recently proposed diagnostic framework for Alzheimer’s disease (AD) sets the biologically defined disease (by amyloid-β and tau) apart from the clinical staging [8]. This underlines that it is pivotal to define which risk one is referring to, since “risk” may refer to an individual’s likelihood of getting a disease such as AD, which does not immediately imply a clinical outcome (here, cognitive impairment or dementia). Yet, “risk” may also refer to the likelihood of developing the adverse clinical outcome (i.e., dementia). Here, we focus mainly on the dementia risk.

The World Health Organization defines risk communication as “an exchange of real-time information, advice and opinions between experts and people facing threats to their health, economic or social well-being” [9]. The purpose of risk communication is to enable individuals to make informed decisions and take action to protect themselves. Because of the influence that perceptions of individualized risk are expected to have on people’s health- and disease-related behavior, the provision of risk information is an essential aspect of any health prevention effort [10]. Thus, risk communication is a crucial step in tailored prevention strategies of dementia incidence.

However, communicating risk is complex and therefore challenging for clinicians [11, 12]. First, the concept of risk is difficult for patients to comprehend and for physicians to explain [12–17]. Second, risk disclosure is an intervention by itself, because of its potential impact on psychological and mental health of the individual [18] and on decisions that are made for example about the future, employment, and living arrangements, and about who to share these results with. Still, knowing your individual potential for prevention and being able to take action through personalized, multi-domain, risk reduction prevention programs are considered important benefits [19, 20]. In addition, there is currently no single risk prediction tool that is fully validated or recommended as the golden standard for clinical practice.

How to communicate dementia risk depends on the context. Within the context of trial enrollment, disclosure of biomarker evidence and hence dementia risk can be warranted and risk disclosure is then embedded in the protocol [17]. In the clinical context, increasing numbers of individuals without cognitive impairment are seeking care [21, 22], and express a need for information, guidance, and practical advice, although they vary in specific information needs and preferences [23–26]. The envisioned BHSs are aimed at providing meaningful answers to this growing demand. These individuals experience a subjective cognitive decline (SCD) [27] or functional cognitive disorders [22] or are just concerned about cognitive decline and/or their brain health and want to preserve their cognitive performance as long as possible. These individuals represent the target population of BHSs (Altomare et al., [3]) and could be subjected to prediction modeling to inform their individualized dementia risk, although these models are not perfect and risk communication remains challenging [28].

To this background, communication strategies should maximize the desired impact of risk information on individuals’ understanding of their health/disease status and dementia risk perception and minimize potential harms. This paper aims to provide an overview of different perspectives, available evidence, and practice recommendations regarding optimal strategies for communicating dementia risk and identify the next steps in the development of an evidence-based risk communication protocol.

## Perspectives on communicating dementia risk

### Ethical perspective

Individuals have a right to know or not know their dementia risk [29]. Whether someone wants to know and how it impacts them is very personal [30]. Among potential personal benefits are a reduction in feelings of uncertainty and anxiety, enhanced preparedness for the future, and improved quality of life [31–34]. On the other hand, facing a high probability to develop dementia can have negative psychological effects, including stress, depression or even suicidal ideation, and affect sense of self, future, and perception of memory [31, 35]. In addition, sharing personal risk information with others could lead to stigmatization as well as social, professional, and legal discrimination. While (inter)national agreements protect genetic privacy and prohibit discrimination, these may not apply to biomarker-based risk, nor address protections for long-term care insurance [36]. Nonetheless, from an ethical perspective, it is questionable whether these are reasons *not* to communicate dementia risk, especially when individuals prefer to know.

Another important argument in the context of provision of dementia risk information is actionability. While some argue that there may be a lack of actionability in the absence of a disease-modifying treatment for AD [18, 37], others point out that learning their likelihood of developing dementia empowers individuals to shift priorities or try to reduce risk through (other) preventive actions, for example controlling modifiable risk factors such as hypertension [1], or adopting a healthier lifestyle [31, 38]. Knowing ones’ risk could thus meaningfully contribute to a worthwhile set of options. It is important to educate, prepare and counsel individuals on all that is known and still uncertain [39], so they can make informed decisions about knowing their dementia risk and utilize their right to self-determination, while they still can.

### Clinician perspective

When facing a risk, proper decision-making by the individual about preventive/protective action requires proper understanding and conceptualization of this risk. Clinicians must explain the medical problem (i.e., dementia), the magnitude of the risk, and its implications in a way that is readily understandable by lay people. Here, we can learn from cancer genetics, with a longer history in risk communication.

In Mendelian, autosomal dominant conditions with full penetrance, the risk of developing the disease corresponds to the risk of inheriting the mutation, i.e., 50% for each child of an affected patient, which is easily understood as the outcome of flipping a coin. This applies to some Mendelian forms of AD (e.g., *Presenilin* mutations). Conveying a risk becomes slightly more complex when penetrance is incomplete (e.g., *BRCA* mutations), say 70%, producing a disease risk of 35%. Here, geneticists will convey a finer analogy, like picking one out of a hundred marbles from a bag, 65 being green (no disease) and 35 red (disease). Next are moderate-penetrance genes like *CHEK2*, causing a significant but limited increase in breast cancer risk. This compares with some moderately penetrant Alzheimer’s genes, including *APOE-ε4*. In families with many affected, other factors than the *CHEK2* mutation are also at play. Hence, pre-symptomatic testing in unaffected at-risk relatives might carry false alarm if the mutation is found to be present (because the associated cancer risk is limited), and false reassurance if the mutation is found to be absent (because the residual risk remains increased over population risk) [40]. The analogy of picking one out of a hundred marbles with different colors could also be used to explain the risk of developing dementia associated with moderately penetrant Alzheimer’s genes, including these false cases.

In hereditary breast and ovarian cancer, pre-symptomatic genetic testing allows for targeted prevention [41]. However, depending on the *magnitude* of the risk, one might weigh the potential benefits and harms of preventive options differently. For example, considering preventive mastectomy with a *BRCA1* mutation versus *CHEK2*. This highlights how effective risk communication is essential, especially when weighing pros and cons of preventive options. With the advent of effective pre-symptomatic strategies in AD, we should learn from the experience learned in genetic counseling in cancer.

### Societal/public perspective

AD is a major healthcare concern for many people [42]. Having been asked to choose the one disease they were most afraid of from a list of seven, respondents in a public opinion survey most frequently identified cancer, followed by AD (about one quarter). Interestingly, there was a considerable public interest in pre-symptomatic diagnostic testing. Asked individuals whether they want to take a medical test which would tell them whether they would develop AD, a plurality in all countries responded that they would be “very likely” or “somewhat likely” to get such test, ranging from 51% in Germany to 78% in Poland. However, there’s also evidence that people’s preferences for knowing their AD status or dementia risk decrease when they have had the possibility to think about the consequences of receiving such information [43].

In light of the changing definitions of AD, which could lead to differences in understanding and miscommunication [44], special attention should be paid to raise awareness in the general public about the spectrum of AD from the asymptomatic to the dementia stage. Similarly, prevention messages should be included in campaigns targeting the general public and at-risk populations. depending on the structure of local health care provision and local opportunities.

## Evidence on communicating dementia risk

Pending evidence on (the effects of different) strategies for dementia risk communication from the envisioned BHSs, we can learn from evidence gathered within the memory clinic setting, the research setting including clinical trials and prospective studies, and the context of brain research registries.

### Memory clinic setting

People present at memory clinics with symptoms or problems in daily life. This is different from the envisioned BHSs mainly aimed at asymptomatic individuals. Yet, memory clinic experience, particularly with regards to MCI, is informative for BHSs [45]. A recent randomized clinical trial (RCT) among individuals with mild cognitive impairment (MCI), showed that receiving information on one’s amyloid-PET status did not improve understanding of the MCI diagnosis nor the capacity to cope with the uncertainty inherent to that diagnosis [46]. Still, the recently published practice guideline by the American Academy of Neurology (AAN) states that an accurate diagnosis of MCI is important, especially to discuss the prognostic possibilities, i.e., risk of dementia [47]. In addition, recommendations were published on how to communicate about amyloid positron emission tomography (PET) results and prognostic information with MCI patients [48]. Nonetheless, communicating the MCI label remains challenging for clinicians [11], and few provide specific or personalized information on the dementia risk to MCI patients [12].

One of the reasons for being reluctant to sharing prognostic information is the apparent lack of individualized risk information for MCI patients, leaving clinicians at best to provide patients with a “fifty-fifty probability.” Recent evidence however illustrates that in MCI, prediction models with good accuracy, calibration, and generalization allow an individualized prognosis based on biomarker evidence [49]. Even when these models still warrant prospective clinical validation, their clinical applicability is at the horizon. On a group level, biomarkers are also predictive of incident dementia in cognitively unimpaired individuals who present with subjective cognitive decline (SCD) at the memory clinic, although it should be noted that at least half of biomarker positive individuals with SCD does not progress to dementia within 5 years [50]. For this population, individualized risk models are not yet ready for implementation, since their external validation is suboptimal [28]. In short, it is not easy to convert biomarker findings to an actual specific and personalized dementia risk. Clinicians’ reluctance in sharing this information is therefore appropriate. To this background, considering the BHS target group of cognitively unimpaired individuals, it is questionable whether we currently have the data available to derive reliable dementia risk estimates. Risk communication can only be as good as the models it is based on, and clearly more work needs to be done.

### Research setting

To identify individuals at higher risk of developing AD or dementia, *APOLIPOPROTEIN (APOE)* and amyloid PET testing are being incorporated in clinical (drug) trials and prospective studies as screening tools (e.g., Alzheimer’s Prevention Initiative Generation Program [51]; Anti-Amyloid Treatment in Asymptomatic Alzheimer’s Disease (A4) Study [52]; AMYPAD Diagnostic and Patient Management Study [53]). Although the disclosure of *APOE-ε4* carriership and amyloid PET positivity have distinct implications, trial protocols have adopted similar recommendations regarding the disclosure of *APOE* and amyloid PET test results (Supplement A in Supplementary Material).

A number of studies have been carried out to assess the safety of disclosing such AD biomarker test results to cognitively unimpaired individuals in the context of trials (Table 1) [31, 32, 54–61]. Most research has shown that disclosure of Alzheimer biomarker results (after pre-test education and psychological screening) does not lead to short-term negative psychological consequences (Table 1). However, it may not always be possible to extrapolate results from highly motivated individuals taking part in research to a more general population. Furthermore, none of the existing disclosure protocols have dealt with actual risk communication, which would imply the provision of the precise magnitude of the risk of developing dementia, considering a specific timeframe (e.g., risk within a number of years). Moreover, additional research is needed to examine the broader implications, both beneficial and harmful, of living with risk, and the impact over longer periods of time.

**Table 1:** An overview of studies investigating the impact of disclosure of APOE and Amyloid PET test results to research participants

Publication	Project/study name	Type of study	Disclosure of	Main finding(s)
Green et al. 2009 [54]	The Risk Evaluation and Education for Alzheimer’s Disease (REVEAL) Study	RCT	*APOE*	No differences between the two groups (disclosure *vs* no disclosure) in changes in time-averaged measures of anxiety, depression, or test-related distress (measured at 6 weeks, 6 months, and 1 year). The *ε4*-negative subgroup had a significantly lower level of test-related distress than did the *ε4*-positive one.
Chao et al. 2008 [55]	REVEAL	RCT	*APOE*	Participants who learned they were ε4 positive were significantly more likely than ε4 negative participants to report AD-specific health behavioral change 1 year after disclosure.
Bemelmans et al. 2016 [31]	N/A	Systematic review	*APOE*	In cognitively unimpaired research participants with a first-degree relative with AD, disclosure of *APOE-ε4* positivity does not lead to elevated anxiety and depression levels. It does increase test-related distress. It results in behavioral changes concerning insurance and health.
Langlois et al. 2019 [56]	Alzheimer’s Prevention Initiative Generation Program	RCT	*APOE*	Standard protocol for disclosure is reported. Analyses have not been published yet.
Harkins et al. 2015 [57]	Anti-Amyloid Treatment in Asymptomatic Alzheimer’s Disease (A4) Study.	Modified Delphi study to develop consensus on best practices	Amyloid PET	Standard protocol for disclosure is reported.
Burns et al. 2017 [58]	University of Kansas Alzheimer’s Prevention through Exercise [APEX]	RCT	Amyloid PET	Depressive symptoms were stable throughout the visits and not different between groups (elevated vs non-elevated amyloid). Anxiety symptoms peaked at a low level on the day of disclosure in the “elevated” group but were not sustained at 6 weeks or 6 months. Individuals with elevated amyloid had slightly higher total levels of test-related distress compared with the non-elevated amyloid group at 6 weeks and 6 months post-disclosure.
Largent et al. 2020 [32]	Study of Knowledge and Reactions to Amyloid Testing (SOKRATES) recruiting participants from the A4 and Longitudinal Evaluation of Amyloid Risk and Neurodegeneration (LEARN) trials	Observational study	Amyloid PET	Participants generally understood that an “elevated” amyloid PET scan result means increased but presently unquantifiable risk of developing AD dementia. Participants who received an “elevated” result often wanted more information regarding the result. An “elevated” result sparked negative emotions that decreased but did not entirely dissipate with time, but did not lead to extreme distress. Support the safety of disclosing amyloid imaging results to cognitively unimpaired persons following pre-test assessments of knowledge and psychological well-being. Participants who received an “elevated” result reported contemplating and making changes to health behaviors and future plans to a greater extent. Participants with elevated brain amyloid viewed the amyloid PET scan result as a serious, sensitive piece of health information. Irrespective of their brain amyloid status, participants were mindful that their amyloid PET scan result had implications for themselves and also for others.
Grill et al. 2020 [59]	A4 study and LEARN trials	Observational study	Amyloid PET	Participants in the elevated amyloid group, compared with participants who learned that they had a not elevated amyloid result, were not more likely to experience short-term increases in depression, anxiety, or suicidality
Wilde et al. 2018 [60]	N/A	Systematic review	Amyloid PET	The sparse data available suggest that disclosure of amyloid PET results has a low risk of psychological harm in the context of clinical trials, whereas both participants and professionals seem to support disclosure. More research is needed about the psychological impact of PET disclosure, and the predictive value of results at an individual level. Communication materials and strategies to support disclosure of amyloid PET results should be further developed and prospectively evaluated.
Kim and Lingler 2019 [61]	N/A	Systematic review	Amyloid PET	Provides important early insights into the psychological safety of disclosing amyloid imaging results to cognitively normal persons. Highlights the need for rigorously designed studies that address social and behavioral outcomes and extend to symptomatic populations.

### Brain research registries

Here, we present three examples of brain research registries, aiming to catalyze trial enrolment of individuals in the earliest disease stages. The success of these registries reflects the high interest in brain research, prevention, and brain health in the general community.

#### The “BBDPS Study: a study on risk factor disclosure” and its associated registry

The registry associated with the Barcelonaβeta Dementia Prevention Study (BBDPS) aims to recruit individuals with SCD or MCI from the general population (Supplement B in Supplementary Material). People were invited to register if they “were feeling changes in their memory or cognitive status.” The study’s schematic is shown in Figure B1 in Supplementary Material. The registry contributes to a highly efficient recruitment strategy, with screening failure rates much lower than standard rates. Moreover, the BBDPS-registry shows value in light of BHSs since ongoing (prevention) studies can be offered to more than 50% of the registered individuals.

In a first evaluation of the emotional impact of disclosing personal risk estimates, the BBDPS-study showed that disclosing the 5-year dementia risk to cognitively unimpaired participants in a research setting increases neither feelings of depression nor anxiety, hence can be considered safe (Table B1 and Figure B2 in Supplementary Material).

#### The Swiss Brain Health Registry

The Swiss Brain Health Registry (www.bhr-suisse.org) aims to facilitate access to research programs to persons who wish to contribute to research on AD and memory-related diseases. The registry is open to all persons aged 50 and over. Once participants sign the informed consent, they are in the registry and can be contacted by researchers from one of the participating Swiss memory clinics (Geneva, Lausanne, Fribourg, St. Gallen or Lugano) and offered the opportunity to participate in a study. The website offers generic advice on lifestyles for a healthy brain, but the Swiss Brain Health Registry does not provide registrants with information on their personal dementia risk.

#### The Dutch Brain Research Registry

The Dutch Brain Research Registry (*Hersenonderzoek.nl* in Dutch) [62] was set up in 2017 with the aim to accelerate recruitment of participants for current and future clinical brain disease studies in the Netherlands. To date, over 20.000 participants signed up (58±11 years old, 78% female). Using their personal online portal, registrants provide demographic information, medical history, family history of dementia, medication and substance use, and lifestyle information. Prescreening of registrants for studies/trials is solely based on this self-reported information. For this reason, the Dutch Brain Research Registry chose not to provide registrants with any information on their personal risk for developing AD and/or dementia.

Between January and June 2019, five focus groups were organized (each 3 to 8 participants, total *n* = 28) to explore registrant experiences, including motivations for registration. In addition to altruistic reasons (contribute to science/society) and family-related reasons (brain disease runs in family), registrants often reported that receiving information about their brain health and gaining insight in improving or maintaining their own brain health were important reasons for registration. These findings indicate that many of the registrants of a low-threshold, online research registry like the Dutch Brain Research Registry are representative of the target population for the envisioned BHSs.

## Practical recommendations on risk communication

### How to communicate risk

Table 2 displays nine practical recommendations on how to communicate about dementia risk, as synthesized from available guidelines and evidence in the oncology field [14, 63, 64]. The online tool ADappt (www.ADappt.health) encompasses a dementia risk calculation tool, including a communication sheet taking into account these recommendations [65].

**Table 2:**
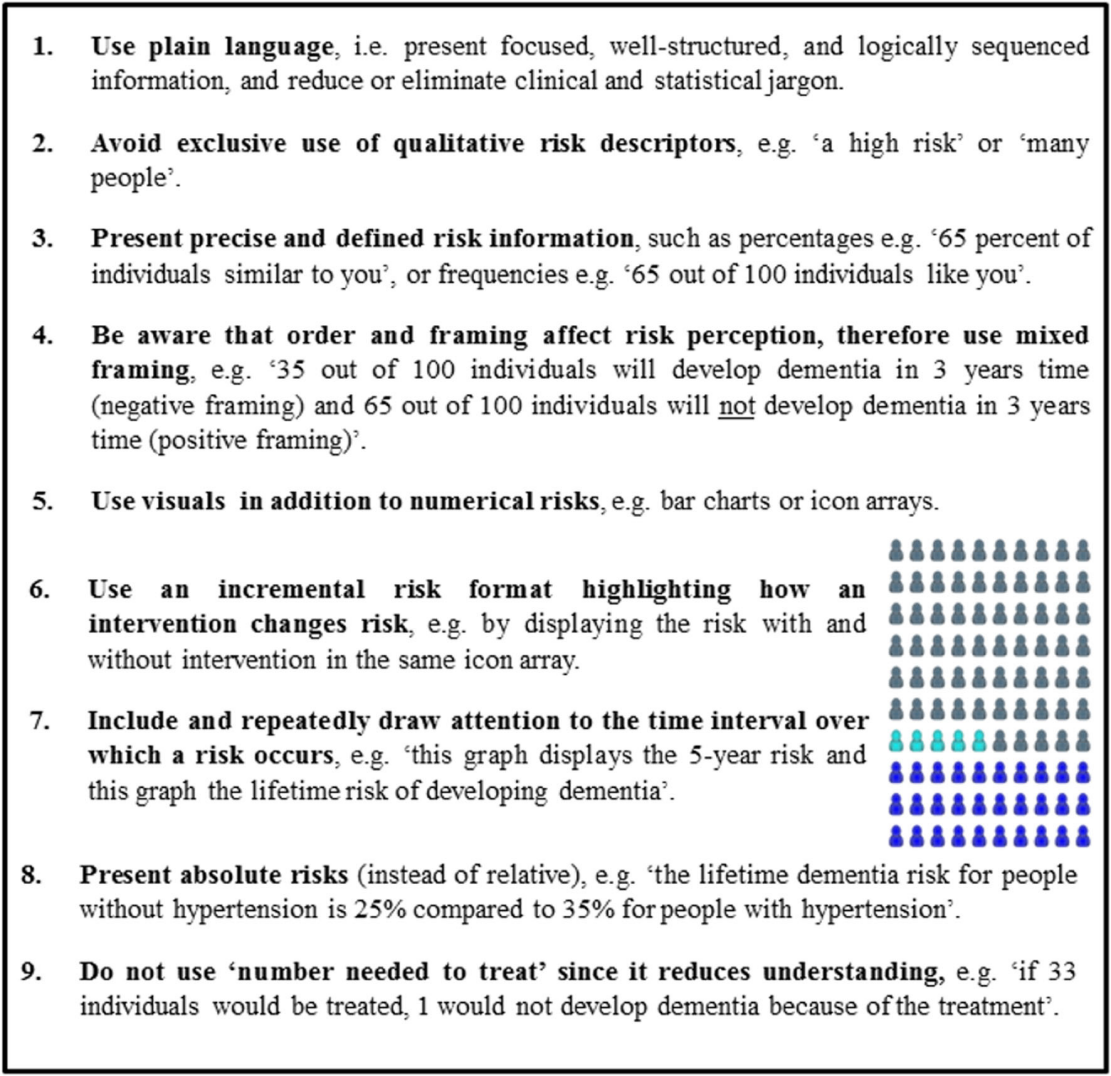
Practice recommendations for communicating risk in BHSs

### Managing expectations: communicating uncertainty and shared decision-making

Risk profiling is about probabilistic statements regarding the individual's future, and therefore uncertainty is inherent [66]. Even when biomarker testing confirms a disease diagnosis, it often does not provide certainty about disease severity or its course [67]. Moreover, high-quality data are sometimes not available and even when they are, these data can have multiple interpretations or be contradictory, and risk models are never perfect [28]. Hence, communication about uncertainty is an integral aspect of risk disclosure, and managing expectations beforehand is important to inform individuals that this process will not necessarily result in a reduction of uncertainty [48, 68]. An approach for uncertainty communication has been proposed [69], which comprises three steps: (1) normalizing uncertainty, by acknowledging individuals' wish for more certainty while explaining that uncertainty is unfortunately inherent to the situation; (2) addressing the individual’s emotions regarding uncertainty by acknowledging that it is unpleasant not to know things; and (3) stimulating individuals to focus on living in the here and now instead of dwelling on the uncertainty, thereby helping them to cope with uncertainty.

Thus, risk communication is not simply about disclosing test results (*i.e.*, one-way information provision), but also about what individuals and their relatives want to know and when (i.e., a two-way information exchange). Especially since individuals might weigh the potential harms and benefits of knowing their dementia risk differently, depending on their values, needs, and situation [25]. A process of shared decision-making (SDM) [70] offers a way to incorporate evidence and the health care professional’s expertise, as well as the individual’s preferences into decision-making about pursuing biomarker testing for AD and/or knowing their dementia risk [71, 72]. Four steps are proposed: (1) informing the individual that a preference-sensitive decision is to be made; (2) explaining the options including pros and cons; (3) discussing what is important for the individual in his/her situation; and (4) discussing the individual’s preferred role in decision-making and make a decision. In general, the SDM approach has been shown to encourage health-promoting behaviors, reduce inappropriate or unnecessary use of care, and improve patient and clinician satisfaction [73, 74].

## Discussion

We reviewed and synthesized current evidence to formulate practice recommendations for communicating dementia risk. Based on evidence in the field of oncology, risk communication should be based on a process of shared decision-making, taking into account that risk implies, by definition, uncertainty. Best practice recommendations for risk communication include the use of absolute risks, visual displays, and time frames.

In memory clinics, patients present with signs and symptoms. Still, even in that context, there is room for improvement in risk communication [24]. Some experience is also gained in the trial setting, where protocols for disclosure of *APOE* and amyloid test results and dementia risk have been developed, and studies show that biomarker disclosure is not harmful in the short term [32, 59, 60]. But often this disclosure only infers the explanation that these biomarker findings *can be viewed as a risk factor* for dementia. They do not specify the time frame or the precise magnitude of the risk, nor do they relate this risk to the general risk of dementia (without knowledge on biomarkers) or the risk based on the individual’s full risk profile (e.g., incorporating cardiovascular factors). Yet, an increasing number of individuals want to know their risk and specifically its meaning [25, 75, 76].

In other fields, such as oncology, quite a lot of research has been done on the optimal way to convey risks. A first attempt to integrate these recommendations in risk communication based on biomarker-findings in patients presenting at a memory clinic with MCI is www.ADappt.health [65]. However, risk communication is not only about *how* to communicate, but also about *what* to tell. The step to executing this type of risk communication in cognitively unimpaired individuals, the target population of BHSs, is quite large. Although findings at group level clearly endorse the predictive value of biomarker findings, these same findings also illustrate large heterogeneity in disease course, and therefore translation to the individual level is limited. It takes a long time from biomarker abnormality to onset of dementia, and this explains why large cohorts with long duration of follow-up are needed.

### Challenges

Evidence about the effectivity of specific risk communication strategies mainly stems from data collected in other medical settings, such as oncology. Some important differences warrant caution when translating that evidence to the field of AD and dementia. In oncology, patients with a given cancer diagnosis are presented with evidence-based treatment options and accompanying known probabilities of survival and other harms and benefits. In comparison, in AD, we are talking about evidence of disease, based on biomarkers, to predict likelihood of developing dementia in the future. These differences emphasize the need for more specific research on risk communication in the context of AD and the implementation of a precision approach to dementia prevention.

In general, risk information is difficult to convey, and for at-risk individuals to understand [77, 78]. It may be even more difficult in dementia, as no fixed events define its onset [79]. As an additional barrier, the cognitive impairment that is inherent to a neurodegenerative disease such as AD could hamper communication and understanding. A particular challenge at this stage is that a lot of people in the general public use the terms AD and dementia interchangeably and are not aware of the new definition of AD as the underlying disease which may cause dementia at a later stage [44, 80]. There is a lot of (linguistic) confusion -also among professionals- making it harder to define optimal strategies to clearly communicate risk.

Finally, decisions about whether and how to pursue diagnostic testing and communicate risk estimates as part of a nation's package of healthcare benefits might also depend on the national structure of health care provision and local opportunities, since there is a wide variety in healthcare benefits/reimbursement systems among countries.

### Future research directions

More evidence is warranted about the impact of dementia risk communication, to be able to adequately inform, make (shared) decisions, and manage expectations. More specifically, we need to systematically evaluate the relative merits of different approaches/strategies to risk communication on outcomes in three domains: (i) the cognitive domain, reflecting peoples’ risk perception and understanding, such as accuracy in answering questions related to probabilistic information; (ii) the affective domain, concerning the psychosocial impact, including stigma, and individuals’ preferences for or satisfaction with the communication strategy; and (iii) the behavioral domain, people’s intentions to change behavior, or their actual actions, such as lifestyle modifications.

Furthermore, research should be directed at identifying those factors that influence individuals’ reactions to getting to know their dementia risk, since differences between individuals may moderate the relation between risk communication and relevant outcomes. Hence, a one-size-fits-all approach does not work. This is especially relevant in light of cultural differences and minority groups, and one's educational background. Insight into moderating factors and individual differences could stimulate the adoption of a truly personalized, tailored approach to dementia prevention, taking into account the individual’s characteristics and personal needs, preferences, values, and situation.

Next, based on that evidence, we should develop an evidence-based dementia risk communication protocol. Although evidence about the impact of different strategies on relevant outcomes could form a strong base, input from health care professionals and the target population will be necessary to align perspectives and gain acceptance among stakeholders, which support the implementation of risk communication best-practices. Finally, (e-)tools are warranted to support professionals in communication and promote adherence to the communication protocol.

### Conclusion

With the growing knowledge of AD and availability of biomarkers on the one hand, and the increasing focus on prevention strategies on the other hand, there is a growing demand to know one’s risk of dementia in very early stages. This demand will even further increase with the upcoming of blood-based biomarkers and disease-modifying treatments. Communicating about the risk of developing dementia is thus crucial, yet challenging, because of the current lack of evidence on *what* to tell on an individual level (i.e., the actual risk), and on *how* to optimally communicate about risk in a way that maximizes the desired impact of this information and minimize its harms. Available evidence suggests that risk communication should be precise and include the use of absolute risks, visual displays, and time frames, be based on a process of shared decision-making, and address the uncertainty inherent to any probability. Next steps required for the development of an evidence-based BHS protocol for dementia risk communication include the systematic evaluation of the relative merits of different strategies to risk communication on affective, cognitive, and behavioral outcomes, with a special focus on individual differences.

## Supplementary Information

**Additional file 1.** Supplement A. Summary of recommendations on how to disclose test results for dementia risk assessment in context of clinical trials. Supplement B. The Barcelonaβeta Dementia Prevention Study (BBDPS) and its associated registry. Figure B1. Schematic representation of the BBDPS. Table B1. Sample characteristics of the BBDPS participants at baseline visit. Figure B2. Impact of dementia risk disclosure on 69 low-risk versus 20 high-risk individuals with SCD from the BBDPS.

## Data Availability

The data that support the findings of the BBDPS of are available from the corresponding author Carolina Minguillon, upon reasonable request.

## Abbreviations

AAN: American Academy of Neurology
AD: Alzheimer’s disease
APOE: APOLIPOPROTEIN
A4: Anti-Amyloid Treatment in Asymptomatic Alzheimer’s Disease Study
BBDPS: Barcelonaβeta Dementia Prevention Study
BHS: Brain Health Services
CES-D: Center for Epidemiologic Studies Depression Scale
MCI: Mild cognitive impairment
PET: Positron emission tomography
SCD: Subjective cognitive decline
SDM: Shared decision-making
STAI: State-Trait Anxiety Inventory
