# Supplementary material for: Dementia risk communication. A user manual for Brain Health Services—part 3 of 6
Source: Alzheimers Res Ther. 2021 Oct 11;13:170. doi: 10.1186/s13195-021-00840-5 (PMC8507171; doi:10.1186/s13195-021-00840-5)
Supplement: Supplementary file 1 — Additional file 1. Supplement A. Summary of recommendations on how to disclose test results for dementia risk assessment in context of clinical trials. Supplement B. The Barcelonaβeta Dementia Prevention Study (BBDPS) and its associated registry. Figure B1. Schematic representation of the BBDPS. Table B1. Sample characteristics of the BBDPS participants at baseline visit. Figure B2. Impact of dementia risk disclosure on 69 low-risk versus 20 high-risk individuals with SCD from the BBDPS. [file 13195_2021_840_MOESM1_ESM.docx]

**Dementia risk communication.**

**A user manual for Brain Health Services – Part 3 of 6**

Leonie N.C. Visser, PhD; Carolina Minguillon, PhD; Gonzalo Sánchez-Benavides, PhD; Marc Abramowicz, MD, PhD; Daniele Altomare, PhD; Karine Fauria, PhD; Giovanni B Frisoni, MD; Jean Georges, BA; Federica Ribaldi, PhD; Philip Scheltens, MD, PhD; Jetske van der Schaar, MA; Marissa Zwan, PhD; Wiesje M. van der Flier, PhD; José Luis Molinuevo, MD, PhD on behalf of the *European Task Force for Brain Health Services*

**SUPPLEMENTARY METERIAL**

**Supplement A**. Summary of recommendations on how to disclose test results for dementia risk assessment in context of clinical trials.

**Supplement B**. The Barcelonaβeta Dementia Prevention Study (BBDPS) and its associated registry.

**Figure B1**. Schematic representation of the BBDPS.

**Table B1**. Sample characteristics of the BBDPS participants at baseline visit.

**Figure B2**. Impact of dementia risk disclosure on 69 low-risk versus 20 high-risk individuals with SCD from the BBDPS.

**Supplement A – Summary of recommendations on how to disclose test results for dementia risk assessment in context of clinical trials**

We synthesized the following recommendations for disclosure of APOE- and amyloid PET status in the context of clinical trials (mainly extracted from [1, 2, 3]):

1. Before screening, provide potential participants with educational brochures in which the main points of the study, the meaning of the test and test results are covered in plain, understandable language.
2. At screening, assess the participant’s emotional status (to assess eligibility/preparedness to receiving the result) and the understanding of the study/test/consequences of the result using the same language as in the study’s brochure.
3. In the case of PET, the scan should be performed on a separate day, i.e., different from the educational/screening session.
4. At disclosure, performed on a separate day from blood draw/scanning, review the educational content and participant’s understanding, and assess emotional status before proceeding with disclosure. For PET results disclosure, positivity is explained as having a higher risk, without specification of the actual risk.
5. Establish a minimum number of follow-up visits (telephonic, in- person) to assess the possible emotional impact of disclosure and arrange additional monitoring and follow-up for participants showing distress.

**Supplement B – The Barcelonaβeta Dementia Prevention Study (BBDPS) and its associated registry**

The Barcelonaβeta Brain Research Center (BBRC) implemented the Barcelonaβeta Dementia Prevention Study (BBDPS) in 2018 including an associated registry aiming at recruiting SCD and MCI individuals.

BBDPS was presented at a press conference (May 16^th^, 2018). The study and its registry were launched and general population were invited to register in the study web page if they “were feeling changes in their memory or cognitive status”. The web-based selection system is designed as to allow the establishment of a registry containing the a priori eligible persons (*a priori* IN) interested in participating in the study, using an algorithm designed to include participants that meet certain criteria (i.e. age (60-80 y.o); >3 SCDplus features [4, 5]; availability). Those not fulfilling the criteria are annotated as *a priori* OUT. To examine the efficiency of the registry in getting the target population, we implemented a simple validation analysis: 232 persons were randomly chosen from the registry, (168 *a priori* IN and 64 *a priori* OUT). A neuropsychologist called each of them to evaluate their cognitive status, applying the same SCDplus questionnaire that was designed for the web (among other questions). Using the same questionnaire, but being able to further examine each of the participants‘ answers by a professional (since the individual is able to verbally justify their answers), enabled to take into account a clinical opinion for the outcome of the SCDplus questionnaire. Thus, an *a priori* eligible status could change after the phone examinations to OUT and, conversely, an *a priori* OUT status could change to IN after the telephonic interview. Out of 168 *a priori* IN, 137 (80%) continued as IN. Nevertheless, a dozen of those still considered as IN have been excluded from the *a priori* IN registry after further examination. This is mainly because the further analysis involved certain clinical criteria (e.g. illnesses non-compatible with the study) and MRI eligibility. Out of 64 *a priori* OUT, 55 (85%) continued as OUT, while the remaining nine were re-considered as IN, after the phone call and professional evaluation.

**Figure B1**. Schematic representation of the BBDPS.


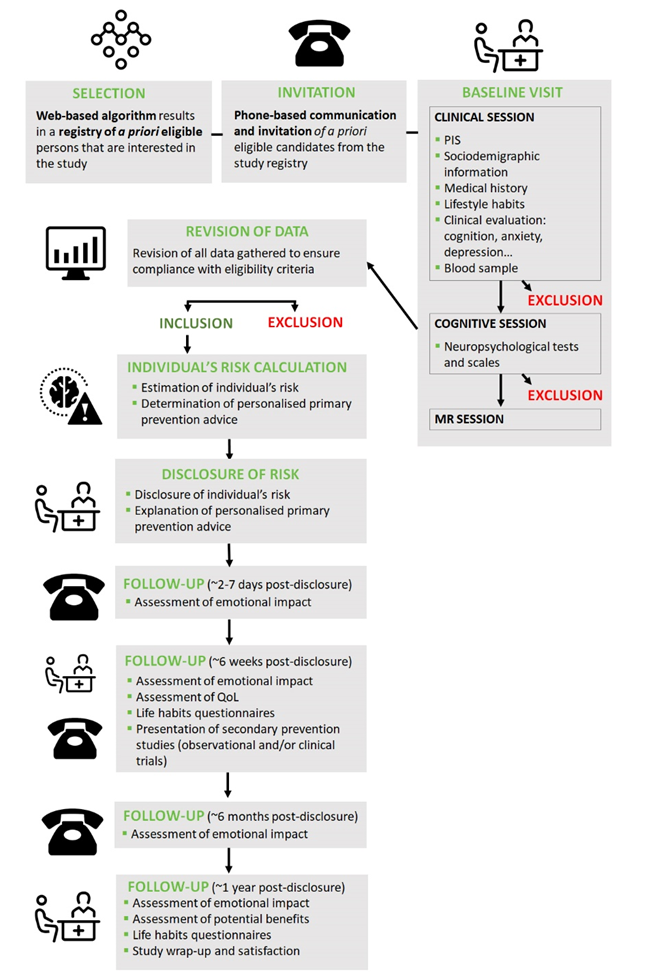


*A priori* IN individuals gathered from the study’s registry, were contacted on the phone to schedule an in-person baseline visit and sent by email the study’s brochure, in which the study, the main risk factors for dementia, how their specific risk would be calculated and how to interpret this risk estimates were explained in plain language. At baseline, the neurologist in charge of the visit explained the study: She/he would use the brochure to go through all this information and an *ad hoc* questionnaire was design to assess the participant’s understanding. Once informed consent was granted, the variables (e.g. clinical, sociodemographic, lifestyle, cognitive, dementia blood workup) that feed the risk algorithm [6] as well as the personalized prevention plans and guidelines obtained, were gathered. These initial data obtained also serve for evaluating compliance with inclusion criteria as well as the diagnostic classification of the subject in a subgroup of MCI or SCD. At baseline, the first emotional evaluation of the participant is also performed (and excluded if exceeded established cut-offs). This baseline visit included the following sessions: Clinical session, Cognitive session, Magnetic resonance (MR) session. All data gathered at baseline was reviewed by BBRC researchers to assess the final inclusion of a study participant. Individual risk estimates were calculated and personalized prevention plans selected depending on the participant’s specific risk profile and their diagnostic classification. At an in-person disclosure visit, after assessing the emotional status of the participant, the individual’s risk estimates were disclosed in function of their diagnostic classification followed by an explanation of personalized primary prevention advice. Individuals within the MCI diagnostic category may be offered other research studies and trials they may benefit from, that are undergoing at BBRC or collaborating institutions. After disclosure, a telephonic follow-up was performed between 2 to 7 days to evaluate emotional impact. Further telephonic (or in-person) follow-up was performed ≈6 weeks post-disclosure to evaluate again any possible emotional impact. In addition, depending on their specific risk profile, secondary prevention strategies were discussed and subjects may be invited to participate in ongoing studies (observational and/or clinical trials). Please note that this follow-up visit may also be in-person. Approximately 6 months post-disclosure a telephonic follow-up was performed to evaluate emotional impact. Finally, in a last in-person visit, one year (+/- 1 month) post-disclosure, the possible emotional impact was again assessed as well as the possible benefits derived from the instalment of the personalised prevention plan. As of 30th June 2020, 306 persons have undergone the BBDPS baseline visit. From the ones with disclosed diagnosis (n=223), 17% were MCI and 57% SCD, meaning that nearly 75% of a priori selected individuals were corresponding to the population targeted. In total, 54% of these participants comply with eligibility criteria with ongoing studies at BBRC and 69% were actually enrolled.

**Table B1**. Sample characteristics of the BBDPS participants at baseline visit.

|  | **low risk** | **high risk** | **p-value** |
| --- | --- | --- | --- |
| **n** | 69 | 20 |  |
| **Age, mean (std)** | 64.1 (2.6) | 72.7 (3.2) | <0.001* |
| **Education, mean (std)** | 15.3 (3.9) | 12.9 (4.8) | 0.065 |
| **sex, female, count (%)** | 38 (55.1%) | 10 (50%) | 0.689 |
| **% of risk, mean (std)** | 3.5% (0.4) | 13.7% (5.8) | <0.001* |
| **CES-D, mean (std)** | 8.5 (8.3) | 9.1 (8.2) | 0.796 |
| **STAI-T, mean (std)** | 35.5 (10.5) | 35.0 (9.4) | 0.949 |
| **STAI-S, mean (std)** | 35.3 (12.5) | 35.2 (7.1) | 0.837 |

*Notes*. CES-D, Center for Epidemiologic Studies Depression Scale; STAI, State-Trait Anxiety Inventory; STAI-S, STAI state; STAI-T, STAI trait; std, standard deviation.

In BBDPS, the emotional impact of disclosing personal risk estimates by analysing depressive (Center for Epidemiologic Studies Depression Scale; CES-D[7]) and anxiety symptoms (State-Trait Anxiety Inventory; STAI[8]), as well as test-related distress (adapted from [1]) in 128 BBDPS participants with SCD, with complete data up to the 6-month post-disclosure follow-up visit, was investigated. From these, two extreme groups were formed (n=69 with low risk as those with <4% risk of developing dementia in the next 5 years, n=20 with high risk as those with >10% risk). Group characteristics, including the mean dementia risk, are shown in Table B1 here.

As shown in Figure B2, no group differences (low vs high risk) were found in depressive (A) and anxiety (B) symptoms or in test-related distress (C) across the study’s time-points, indicating that disclosing 5-year dementia risk to cognitively unimpaired participants in a research setting is safe.

**Figure B2**. Impact of dementia risk disclosure on 69 low-risk versus 20 high-risk individuals with SCD from the BBDPS.


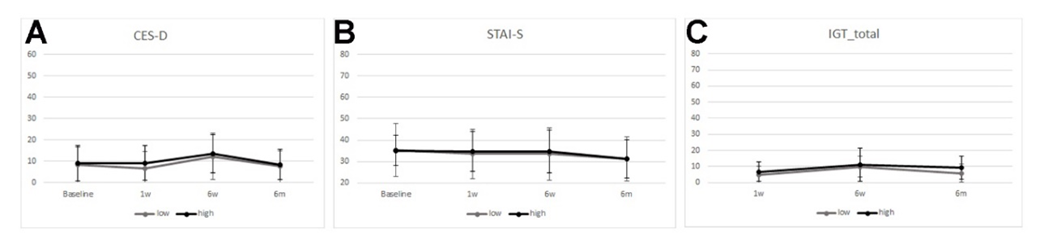


*Notes*. Data at baseline, 1 week, 6 weeks and 6 months post-disclosure on A: depressive symptoms (CES-D) and B: anxiety levels (STAI-State). Graph C displays test-related distress (IGT-AD scale) at 1-week, 6-week and 6-month post-disclosure. Means and standard deviations are shown. Y-axis shows full scale range.

**REFERENCES (Supplementary Material)**

1. Burns JM, Johnson DK, Liebmann EP, Bothwell RJ, Morris JK, Vidoni ED: **Safety of disclosing amyloid status in cognitively normal older adults**. *Alzheimer's & dementia: the journal of the Alzheimer's Association* 2017, **13**(9):1024-1030.
2. Harkins K, Sankar P, Sperling R, Grill JD, Green RC, Johnson KA, Healy M, Karlawish J: **Development of a process to disclose amyloid imaging results to cognitively normal older adult research participants**. *Alzheimers Res Ther* 2015, **7**(1):26.
3. Langlois CM, Bradbury A, Wood EM, Roberts JS, Kim SYH, Riviere ME, Liu F, Reiman EM, Tariot PN, Karlawish J *et al*: **Alzheimer's Prevention Initiative Generation Program: Development of an APOE genetic counseling and disclosure process in the context of clinical trials**. *Alzheimers Dement (N Y)* 2019, **5**:705-716.
4. Jessen F, Amariglio RE, van Boxtel M, Breteler M, Ceccaldi M, Chetelat G, Dubois B, Dufouil C, Ellis KA, van der Flier WM *et al*: **A conceptual framework for research on subjective cognitive decline in preclinical Alzheimer's disease**. *Alzheimer's & dementia : the journal of the Alzheimer's Association* 2014, **10**(6):844-852.
5. Molinuevo JL, Rabin LA, Amariglio R, Buckley R, Dubois B, Ellis KA, Ewers M, Hampel H, Kloppel S, Rami L *et al*: **Implementation of subjective cognitive decline criteria in research studies**. *Alzheimer's & dementia : the journal of the Alzheimer's Association* 2017, **13**(3):296-311.
6. Li J, Ogrodnik M, Devine S, Auerbach S, Wolf PA, Au R: **Practical risk score for 5-, 10-, and 20-year prediction of dementia in elderly persons: Framingham Heart Study**. *Alzheimer's & dementia : the journal of the Alzheimer's Association* 2018, **14**(1):35-42.
7. Radloff LS: **The CES-D Scale: a self-report depression scale for research in the general population.** . *Appl Psych Meas* 1977(1):385-401.
8. Spielberger CD, Gorsuch RL, Lushene R, Vagg PR, Jacobs GA: **Manual for the State-Trait Anxiety Inventory.** . Palo Alto, CA: Consulting Psychologists Press; 1983.
